# Chinese expert consensus on the diagnosis and treatment of malignant pleural mesothelioma

**DOI:** 10.1111/1759-7714.15022

**Published:** 2023-07-17

**Authors:** Qian Wang, Chunwei Xu, Wenxian Wang, Yongchang Zhang, Ziming Li, Zhengbo Song, Jiandong Wang, Jinpu Yu, Jingjing Liu, Shirong Zhang, Xiuyu Cai, Wen Li, Ping Zhan, Hongbing Liu, Tangfeng Lv, Liyun Miao, Lingfeng Min, Jiancheng Li, Baogang Liu, Jingping Yuan, Zhansheng Jiang, Gen Lin, Xiaohui Chen, Xingxiang Pu, Chuangzhou Rao, Dongqing Lv, Zongyang Yu, Xiaoyan Li, Chuanhao Tang, Chengzhi Zhou, Junping Zhang, Hui Guo, Qian Chu, Rui Meng, Xuewen Liu, Jingxun Wu, Xiao Hu, Jin Zhou, Zhengfei Zhu, Xiaofeng Chen, Weiwei Pan, Fei Pang, Wenpan Zhang, Qijie Jian, Kai Wang, Liping Wang, Youcai Zhu, Guocai Yang, Xinqing Lin, Jing Cai, Huijing Feng, Lin Wang, Yingying Du, Wang Yao, Xuefei Shi, Xiaomin Niu, Dongmei Yuan, Yanwen Yao, Jianhui Huang, Xiaomin Wang, Yinbin Zhang, Pingli Sun, Hong Wang, Mingxiang Ye, Dong Wang, Zhaofeng Wang, Yue Hao, Zhen Wang, Bing Wan, Donglai Lv, Jianwei Yu, Jin Kang, Jiatao Zhang, Chao Zhang, Lixin Wu, Lin Shi, Leiguang Ye, Gaoming Wang, Yina Wang, Feng Gao, Jianfei Huang, Guifang Wang, Jianguo Wei, Long Huang, Bihui Li, Zhang Zhang, Zhongwu Li, Yueping Liu, Yuan Li, Zhefeng Liu, Nong Yang, Lin Wu, Qiming Wang, Wenbin Huang, Zhuan Hong, Guansong Wang, Fengli Qu, Meiyu Fang, Yong Fang, Xixu Zhu, Kaiqi Du, Jiansong Ji, Yi Shen, Jing Chen, Yiping Zhang, Shenglin Ma, Yuanzhi Lu, Yong Song, Anwen Liu, Wenzhao Zhong, Wenfeng Fang

**Affiliations:** ^1^ Department of Respiratory Medicine Affiliated Hospital of Nanjing University of Chinese Medicine, Jiangsu Province Hospital of Chinese Medicine Nanjing China; ^2^ Institute of Cancer and Basic Medicine (ICBM) Chinese Academy of Sciences Hangzhou China; ^3^ Department of Chemotherapy Chinese Academy of Sciences University Cancer Hospital (Zhejiang Cancer Hospital) Hangzhou China; ^4^ Department of Respiratory Medicine Affiliated Jinling Hospital, Medical School of Nanjing University Nanjing China; ^5^ Department of Medical Oncology, Lung Cancer and Gastrointestinal Unit, Hunan Cancer Hospital/The Affiliated Cancer Hospital of Xiangya School of Medicine Central South University Changsha China; ^6^ Department of Shanghai Lung Cancer Center, Shanghai Chest Hospital Shanghai Jiao Tong University Shanghai China; ^7^ Department of Pathology Affiliated Jinling Hospital, Medical School of Nanjing University Nanjing China; ^8^ Department of Cancer Molecular Diagnostics Core Tianjin Medical University Cancer Institute and Hospital Tianjin China; ^9^ Department of Thoracic Cancer Jilin Cancer Hospital Changchun China; ^10^ Translational Medicine Research Center, Key Laboratory of Clinical Cancer Pharmacology and Toxicology Research of Zhejiang Province, Affiliated Hangzhou First People's Hospital, Cancer Center Zhejiang University School of Medicine Hangzhou China; ^11^ Department of VIP Inpatient, Sun Yet‐Sen University Cancer Center, State Key Laboratory of Oncology in South China Collaborative Innovation Center for Cancer Medicine Guangzhou China; ^12^ Key Laboratory of Respiratory Disease of Zhejiang Province, Department of Respiratory and Critical Care Medicine, Second Affiliated Hospital of Zhejiang University School of Medicine, Cancer Center Zhejiang University Hangzhou China; ^13^ Department of Respiratory Medicine, Affiliated Drum Tower Hospital Medical School of Nanjing University Nanjing China; ^14^ Department of Respiratory Medicine Clinical Medical School of Yangzhou University, Subei People's Hospital of Jiangsu Province Yangzhou China; ^15^ Department of Radiation Oncology Fujian Medical University Cancer Hospital & Fujian Cancer Hospital Fuzhou China; ^16^ Department of Oncology Harbin Medical University Cancer Hospital Harbin China; ^17^ Department of Pathology Renmin Hospital of Wuhan University Wuhan China; ^18^ Department of Integrative Oncology Tianjin Medical University Cancer Institute and Hospital Tianjin China; ^19^ Department of Medical Oncology Fujian Medical University Cancer Hospital & Fujian Cancer Hospital Fuzhou China; ^20^ Department of Thoracic Surgery Fujian Medical University Cancer Hospital & Fujian Cancer Hospital Fuzhou China; ^21^ Department of Medical Oncology, Hunan Cancer Hospital/The Affiliated Cancer Hospital of Xiangya School of Medicine Central South University Changsha China; ^22^ Department of Radiotherapy and Chemotherapy, Hwamei Hospital University of Chinese Academy of Sciences Ningbo China; ^23^ Department of Pulmonary Medicine Taizhou Hospital of Wenzhou Medical University Taizhou China; ^24^ Department of Respiratory Medicine, the 900th Hospital of the Joint Logistics Team (the Former Fuzhou General Hospital) Fujian Medical University Fuzhou China; ^25^ Department of Oncology, Beijing Tiantan Hospital Capital Medical University Beijing China; ^26^ Department of Medical Oncology Peking University International Hospital Beijing China; ^27^ State Key Laboratory of Respiratory Disease, National Clinical Research Center for Respiratory Disease, Guangzhou Institute of Respiratory Health The First Affiliated Hospital of Guangzhou Medical University(The First Affiliated Hospital of Guangzhou Medical University) Guangzhou China; ^28^ Department of Thoracic Oncology Shanxi Academy of Medical Sciences, Shanxi Bethune Hospital Taiyuan China; ^29^ Department of Medical Oncology The First Affiliated Hospital of Xi'an Jiaotong University Xi'an China; ^30^ Department of Oncology, Tongji Hospital of Tongji Medical College Huazhong University of Science and Technology Wuhan China; ^31^ Cancer Center, Union Hospital, Tongji Medical College Huazhong University of Science and Technology Wuhan China; ^32^ Department of Oncology, the Third Xiangya Hospital Central South University Changsha China; ^33^ Department of Medical Oncology, the First Affiliated Hospital of Medicine Xiamen University Xiamen China; ^34^ Zhejiang Key Laboratory of Radiation Oncology Cancer Hospital of the University of Chinese Academy of Sciences (Zhejiang Cancer Hospital) Hangzhou China; ^35^ Department of Medical Oncology, Sichuan Cancer Hospital & Institute, Sichuan Cancer Center, School of Medicine University of Electronic Science and Technology Chengdu China; ^36^ Department of Radiation Oncology Fudan University Shanghai Cancer Center Shanghai China; ^37^ Department of Oncology Jiangsu Province Hospital and Nanjing Medical University First Affiliated Hospital Nanjing China; ^38^ Department of Cell Biology, College of Medicine Jiaxing University Jiaxing China; ^39^ Department of Medical Shanghai OrigiMed Co, Ltd Shanghai China; ^40^ Department of Oncology Baotou Cancer Hospital Baotou China; ^41^ Department of Thoracic Disease Diagnosis and Treatment Center, Zhejiang Rongjun Hospital The Third Affiliated Hospital of Jiaxing University Jiaxing China; ^42^ Department of Thoracic Surgery, Zhoushan Hospital Wenzhou Medical University Zhoushan China; ^43^ Department of Oncology Second Affiliated Hospital of Nanchang University Nanchang China; ^44^ Department of Pathology Shanxi Academy of Medical Sciences, Shanxi Bethune Hospital Taiyuan China; ^45^ Department of Oncology The First Affiliated Hospital of Anhui Medical University Hefei China; ^46^ Department of Interventional Oncology The First Affiliated Hospital, Sun Yat‐sen University Guangzhou China; ^47^ Department of Respiratory Medicine, Huzhou Hospital Zhejiang University School of Medicine Huzhou China; ^48^ Department of Oncology Lishui Municipal Central Hospital Lishui China; ^49^ Department of Oncology the Second Affiliated Hospital of Medical College, Xi'an Jiaotong University Xi'an China; ^50^ Department of Pathology The Second Hospital of Jilin University Changchun China; ^51^ Senior Department of Oncology The 5th Medical Center of PLA General Hospital Beijing China; ^52^ Department of Radiation Oncology Affiliated Jinling Hospital, Medical School of Nanjing University Nanjing China; ^53^ Department of Respiratory Medicine The Affiliated Jiangning Hospital of Nanjing Medical University Nanjing China; ^54^ Department of Clinical Oncology The 901 Hospital of Joint Logistics Support Force of People Liberation Army Hefei China; ^55^ Department of Respiratory Medicine Affiliated Hospital of Jiangxi University of Chinese Medicine, Jiangxi Province Hospital of Chinese Medicine Nanchang China; ^56^ Guangdong Lung Cancer Institute, Guangdong Provincial Laboratory of Translational Medicine in Lung Cancer Guangdong Provincial People's Hospital, Guangdong Academy of Medical Sciences, School of Medicine Guangzhou China; ^57^ Department of Respiratory Medicine Zhongshan Hospital, Fudan University Shanghai China; ^58^ Department of Thoracic Surgery, Xuzhou Central Hospital Xuzhou Clinical School of Xuzhou Medical University Xuzhou China; ^59^ Department of Oncology, The First Affiliated Hospital, College of Medicine Zhejiang University Hangzhou China; ^60^ Department of Thoracic Surgery The Fourth Hospital of Hebei Medical University Shijiazhuang China; ^61^ Department of Clinical Biobank Affiliated Hospital of Nantong University Nantong China; ^62^ Department of Respiratory Medicine Huashan Hospital, Fudan University Shanghai China; ^63^ Department of Pathology Shaoxing People's Hospital (Shaoxing Hospital, Zhejiang University School of Medicine) Shaoxing China; ^64^ Department of Oncology The Second Affiliated Hospital of Guilin Medical University Guilin China; ^65^ International Cooperative Laboratory of Traditional Chinese Medicine Modernization and Innovative Drug Discovery of Chinese Ministry of Education (MOE), Guangzhou City Key Laboratory of Precision Chemical Drug Development, School of Pharmacy Jinan University Guangzhou China; ^66^ Key Laboratory of Carcinogenesis and Translational Research (Ministry of Education/Beijing), Department of Pathology Peking University Cancer Hospital & Institute Beijing China; ^67^ Department of Pathology The Fourth Hospital of Hebei Medical University Shijiazhuang China; ^68^ Department of Pathology Fudan University Shanghai Cancer Center Shanghai China; ^69^ Department of Internal Medicine The Affiliated Cancer Hospital of Zhengzhou University, Henan Cancer Hospital Zhengzhou China; ^70^ Department of Pathology the First Affiliated Hospital of Henan University of Science and Technology Luoyang China; ^71^ Department of Medical Oncology, Jiangsu Cancer Hospital Nanjing Medical University Affiliated Cancer Hospital Nanjing China; ^72^ Institute of Respiratory Diseases, Xinjian Hospital Third Military Medical University Chongqing China; ^73^ Department of Medical Oncology, Sir Run Run Shaw Hospital Zhejiang University Hangzhou China; ^74^ Department of Radiology Lishui Municipal Central Hospital Lishui China; ^75^ Department of Thoracic Surgery, Affiliated Jinling Hospital Medical School of Nanjing University Nanjing China; ^76^ Department of Oncology, Key Laboratory of Clinical Cancer Pharmacology and Toxicology Research of Zhejiang Province, Affiliated Hangzhou Cancer Hospital, Cancer Center Zhejiang University School of Medicine Hangzhou China; ^77^ Department of Clinical Pathology The First Affiliated Hospital of Jinan University Guangzhou China; ^78^ Department of Medical Oncology, Sun Yat‐sen University Cancer Center, State Key Laboratory of Oncology in South China Collaborative Innovation Center for Cancer Medicine Guangzhou China

**Keywords:** diagnosis, malignant pleural mesothelioma, prognosis, treatment

## Abstract

Malignant pleural mesothelioma (MPM) is a malignant tumor originating from the pleura, and its incidence has been increasing in recent years. Due to the insidious onset and strong local invasiveness of MPM, most patients are diagnosed in the late stage and early screening and treatment for high‐risk populations are crucial. The treatment of MPM mainly includes surgery, chemotherapy, and radiotherapy. Immunotherapy and electric field therapy have also been applied, leading to further improvements in patient survival. The Mesothelioma Group of the Yangtze River Delta Lung Cancer Cooperation Group (East China LUng caNcer Group, ECLUNG; Youth Committee) developed a national consensus on the clinical diagnosis and treatment of MPM based on existing clinical research evidence and the opinions of national experts. This consensus aims to promote the homogenization and standardization of MPM diagnosis and treatment in China, covering epidemiology, diagnosis, treatment, and follow‐up.

## Graphical abstract

Screening, diagnosis, treatment and follow‐up of malignant pleural mesothelioma.

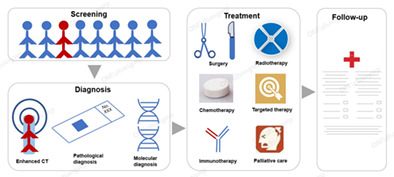

## INTRODUCTION

Mesothelioma is a rare tumor that originates from mesothelial cells in the pleura or other sites, with about 81% on pleura and other sites including the peritoneum, tunica vaginalis, and pericardium.^1^, ^2^ Malignant pleural mesothelioma (MPM) is often diagnosed at advanced stage, making treatment difficult and with poor efficacy. In China, MPM often presents with nonspecific and hidden clinical symptoms, and its histological types are also diverse, leading to low diagnosis and high misdiagnosis rates. With the application of immunotherapy and electric field therapy, the survival of patients has been further improved. In recent years, there has been a significant amount of clinical research conducted globally and regionally on these issues, which has yielded promising results. China has played a crucial role in contributing to this research by providing valuable data and conducting important studies.

Currently, there are several guidelines and consensuses both domestically and internationally that provide guidance for the clinical standard diagnosis and treatment of MPM.^3^, ^4^, ^5^, ^6^ However, there are still many issues that need to be confirmed and standardized in clinical practice, especially in the standardized application of new diagnostic techniques and treatment methods. Therefore, the Mesothelioma Group of the Yangtze River Delta Lung Cancer Cooperation Group (East China LUng caNcer Group, ECLUNG; Youth Committee) organized relevant experts to refer to existing literature evidence and NCCN/ESMO guidelines for in‐depth discussions and exchanges, and ultimately formed expert consensus in order to promote the transformation of new developments in the diagnosis and treatment of MPM into practical clinical benefits.

## INCIDENCE, ETIOLOGY AND SCREENING

**Consensus 1:** The definite diagnosis rate of malignant pleural mesothelioma in China is low, and the misdiagnosis rate is high. It is essential to screen for malignant pleural mesothelioma in high‐risk populations and high‐risk areas **(recommended).**

### Incidence

The latest global cancer burden data released by the International Agency for Research on Cancer (IARC) of the World Health Organization (WHO) showed that in 2020, there were 30 870 new cases of malignant mesothelioma worldwide, accounting for 0.2% of all new cases of malignant tumors globally. The number of deaths was 26 278, accounting for 0.3% of all deaths from malignant tumors worldwide.^7^

In the 2019 China Cancer Registration Annual Report, 487 high‐quality registration offices were selected from 682 cancer data registration offices nationwide, covering a population of 380 million. The detailed description of the cancer disease burden shows that in 2016, there were 583 new cases of mesothelioma in China, including 330 new cases of MPM (International Classification of Diseases [ICD10] code C45.0), with an incidence rate of about 0.86/1 000 000 and a standardized incidence rate (Segi's world standard population) of 0.53/1 000 000. The incidence rate trend showed no significant change. There were 215 deaths for MPM, a death rate of about 0.56/1 000 000 and a standardized incidence rate (Segi's world standard population) of 0.53/1 000 000.^8^ Compared to data released by countries or regions such as Europe and America, the incidence and mortality rates of MPM in China are much lower. The age‐specific incidence and mortality rates of MPM in China increase rapidly after 40 years old, reaching a peak at 80 years old and above.^9^

### Etiology

Asbestos is a mineral that is not inherently toxic, but it is extremely small and almost invisible when floating in the air. It is easily inhaled and deposited in the lungs. Prolonged inhalation of asbestos fibers can cause serious health complications such as lung cancer and MPM.^10^ Asbestos exposure is related to 80% of MPM cases. The latency period from asbestos exposure to the development of MPM is as long as 35–40 years.^11^ Both occupational and nonoccupational asbestos exposure is unsafe, especially for young people and children,^12^ Currently, multiple countries and regions have banned asbestos. However, at the national level, there is a positive correlation between the incidence of mesothelioma caused by asbestos exposure and the social population index.^13^

In addition to asbestos exposure, there are other factors that may lead to the occurrence of MPM. Erionite is a mineral that is commonly associated with occupational diseases in the mining and processing industries, including pleural mesothelioma.^14^, ^15^ Ionizing radiation may also cause mesothelioma to occur.^16^ Based on a large‐scale study of lymphoma patients in the United States who were followed up more than 40 years, MPM is the most common secondary cancer that occurs in some Hodgkin's lymphoma patients who have received mantle field radiation therapy.^17^ Moreover, mutations are also associated with the occurrence of MPM. Studies have found that some patients without asbestos exposure have BRCA associated protein 1 (BAP1) gene mutations or other rare gene mutations.^18^, ^19^

### Screening

For individuals with a family history of MPM and history of occupational asbestos exposure, the risk of developing mesothelioma is 24 times higher compared to those without family history or asbestos exposure (HR = 24, 95% CI: 15–39). This suggests that these people are at high risk and should receive close screening.^20^

Currently, there is no evidence to suggest that low‐dose spiral computed tomography (CT) screening can reduce the mortality rate of MPM in high‐risk populations.^21^, ^22^, ^23^ Therefore, it is not commonly recommended to use low‐dose spiral CT screening for MPM.

Yunnan's Dayao and Zhejiang's Ningbo are two high incidence areas of malignant mesothelioma in China. The epidemiological characteristics of malignant mesothelioma in China are: (1) low age of onset, with an average age of onset of 50 years; (2) higher incidence rate in females and (3) peritoneal mesothelioma is the main type of onset, while pleural mesothelioma is the main type of onset in foreign countries.^24^, ^25^ Retrospective survey data shows that the misdiagnosis rate of MPM in the eastern coastal areas of China is as high as 2/3, which is related to the relative rarity of the disease and insufficient diagnosis.^24^

This indicates that clinical diagnosis of MPM in China is insufficient, with a high misdiagnosis rate, and there is a need to pay attention to screening high‐risk populations and high‐risk areas.

## DIAGNOSIS

**Consensus 2:** Chest and abdominal enhanced CT is currently the preferred imaging diagnostic method for malignant pleural mesothelioma. MRI and PET/CT have their own characteristics and advantages and can be selected for use when conditions permit **(strongly recommended).**

**Consensus 3:** CT‐guided puncture biopsy should be recommended as the standard operation, and ultrasound‐guided puncture biopsy or thoracoscopy should be used as effective supplementary methods in specific situations **(strongly recommended).**

**Consensus 4:** Histopathology is the gold standard for diagnosing malignant pleural mesothelioma, and the pathological report should include histological subtypes and Ki‐67 proliferation index. This includes epithelioid, sarcomatoid, and biphasic types. For epithelioid mesothelioma, the growth pattern should be reported as much as possible in the pathological report. In situ mesothelioma needs to be diagnosed by immunohistochemical detection of BAP1 and/or MTAP loss, and/or by fluorescence in situ hybridization detection of *CDKN2A* homozygous deletion, and fully discussed with thoracic surgeons and radiologists in MDT. The nuclear grading of epithelioid diffuse mesothelioma should be recorded in the pathological report. The degree of cellular atypia, the number of mitotic figures, and the presence or absence of necrosis should also be noted. Common mesothelioma markers include Calretinin, CK5/6, WT‐1, D2‐40, BAP1, MTAP, etc. It is recommended to use at least three mesothelioma markers simultaneously. Sarcomatoid mesothelioma cells do not usually shed into the pleural cavity, and routine pleural effusion cytology is not recommended for this type. In cases where tissue specimens are insufficient or difficult to obtain, it is recommended to use pleural effusion exfoliative cytology to confirm the diagnosis, and to distinguish the cytological features of reactive mesothelial hyperplasia, malignant mesothelioma, and metastatic carcinoma **(strongly recommended).**

**Consensus 5:** Common gene mutations in malignant pleural mesothelioma include *TP53*, *BAP1*, *CDKN2A*, *LATS1/2*, *NF2* and *SETD2*. For young patients without a history of asbestos exposure or a family history of tumors, *BAP1* gene testing is recommended **(strongly recommended).**

### Clinical diagnosis

Malignant pleural mesothelioma is a rare and aggressive disease. Common symptoms include shortness of breath, chest pain, coughing, insomnia, fatigue, loss of appetite, and weight loss. The clinical symptoms of MPM are often more severe than other tumors, and patients with localized lesions may experience significant shortness of breath and chest pain. MPM rarely presents with distant metastasis and related symptoms, and central nervous system metastasis is uncommon, while paraneoplastic syndrome is rare. For individuals with thickening of the pleura, it is recommended to undergo enhanced chest CT examination and pathological or cytological examination to confirm the diagnosis. Monitoring the level of soluble mesothelin‐related peptides may also be helpful, as it may be related to MPM.^26^ Malignant pleural mesothelioma mainly needs to be distinguished from benign pleural diseases and other malignant tumors with pleural metastasis (such as lung cancer, sarcoma, and other solid tumors). Imaging examinations have a suggestive role but are difficult to diagnose. Pathological or cytological examinations are the main methods for differential diagnosis.

### Imaging diagnosis

The accurate staging of MPM requires a combination of imaging examinations and invasive explorations. It can be divided into noninvasive and invasive examinations. Noninvasive examinations include CT, magnetic resonance imaging (MRI), ultrasound, positron emission tomography/computed tomography (PET‐CT), or PET‐MRI. Invasive examinations include pleural biopsy (guided by CT or ultrasound), internal thoracoscopy, surgical thoracoscopy, mediastinoscopy, and laparoscopy.

It is recommended that enhanced CT of the chest and abdomen should be used first for clinical staging. Chest MRI has higher sensitivity for evaluating chest wall, spine, diaphragm or vascular lesions.^23^ Especially for patients who are contraindicated for iodine contrast agents, they can choose chest MRI. PET‐CT is mainly used for staging evaluation of surgical patients. Compared with CT alone, PET‐CT has higher specificity in staging of stage II (77% vs. 100%, *p* < 0.01) and stage III (75% vs. 100%, *p* < 0.01) MPM.^27^ However, another study showed that the sensitivity of PET‐CT for N_1_ stage and T_4_ stage MPM was low (38% and 67%, respectively).^28^

Malignant pleural mesothelioma usually first metastasizes to the mediastinal lymph nodes rather than the hilar lymph nodes. It is difficult to accurately stage the disease using noninvasive examinations. For patients considering surgery, it is recommended to use mediastinoscopy or endobronchial ultrasound to sample and explore the mediastinal lymph nodes.^29^, ^30^

### Pathological diagnosis

Malignant pleural mesothelioma is a heterogeneous tumor, histopathologically including three main subtypes: epithelioid, sarcomatoid, and biphasic. All pathological diagnoses should give the above main pathological subtypes. Pathological detection techniques used for MPM mainly include immunohistochemistry, fluorescence in situ hybridization analysis, and DNA + RNA next‐generation sequencing.

When submitting specimens, it is necessary to provide complete clinical information about the patient, such as occupational exposure history, imaging findings, tumor history, and treatment history. Incomplete clinical information can affect initial diagnosis, specimen processing, sampling procedures, and subsequent auxiliary analysis.

There are various sample types used for diagnosis, including specimens from thoracoscopic, open surgery, CT‐guided core needle biopsy, ultrasound‐guided core needle biopsy, thoracoscopic‐guided thoracic biopsy, fine needle aspiration cytology, and pleural effusion cytology. Pleural biopsy is usually performed through thoracoscopy or percutaneous puncture biopsy guided by CT or ultrasound, which is the main method of obtaining samples. For patients who may undergo surgery, it is recommended to perform single‐port thoracoscopy examination on the potential incision site. Fine needle biopsy and cytological examination of shed cells may have significant sampling bias and often result in false negatives.^31^ The accuracy is low, and fine needle biopsy and exfoliative cytology are not routinely recommended as sample diagnostic basis.

According to the 2021 classification standards for pleural tumors by the World Health Organization (WHO), the histological subtypes of malignant pleural mesothelioma (MPM) mainly include epithelioid, sarcomatoid, and biphasic types, among which the epithelioid type is the most common. To diagnose biphasic MPM, both epithelioid and sarcomatoid components must be present in more than 10%.^32^ The 2021 WHO classification criteria for pleural mesothelioma pathology (fifth edition) are shown in Table 1.

**TABLE 1:** Pathological classification of mesothelioma (fifth edition).

Tumor type	Biological behavior
Diffuse mesothelioma	
Epithelioid mesothelioma	Malignant
Sarcomatoid mesothelioma	Malignant
Biphasic methothelioma	Malignant
Localized mesothelioma	Malignant
Adenomatoid tumor	Benign and prodromal lesions
Well differentiated papillary mesothelial tumor (WDPMT)	Benign and prodromal lesions
Mesothelioma in situ	Benign and prodromal lesions

Malignant pleural mesothelioma is often accompanied by pleural effusion on initial diagnosis. Pleural fluid cytology examination is an easily performed diagnostic procedure and one of the methods for the early diagnosis of MPM. However, the sensitivity of cytology testing is low, and cells of sarcomatoid mesothelioma do not usually shed into the pleural cavity.^33^ Therefore, it is not a routine recommendation to use pleural fluid cytology as a diagnostic basis. However, in patients in which pleural tissue lesions cannot be obtained, if there is a sufficient and representative number of mesothelioma cells, immunohistochemistry and fluorescence in situ hybridization analysis can be performed by preparing cell wax blocks, combined with clinical, imaging, and/or surgical examinations for the diagnosis of MPM. When the cell morphology shows varying degrees of atypia (usually low‐grade) but the malignancy cannot be determined, the term “atypical mesothelial cell proliferation” can be used, but it is not sufficient to diagnose MPM.

Immunohistochemistry can be used to differentiate MPM and its subtypes from other malignant tumors or pleural metastatic tumors, with high diagnostic accuracy and specificity. The main markers supporting the diagnosis of MPM include calretinin, CK5/6, WT‐1, mesothelin, and D2‐40, while the main markers supporting the diagnosis of lung adenocarcinoma include TTF‐1, napsin A, carcinoembryonic antigen (CEA), BerEP4, and claudin4.^33^, ^34^ At least three markers for MPM and three markers for lung adenocarcinoma should be used for differential diagnosis, and the diagnosis should be made by a pathologist with experience in diagnosing MPM. Sarcomatoid mesothelioma does not usually express any typical mesothelioma markers, and positive keratin may be helpful in diagnosing sarcomatoid mesothelioma. When differentiating from other metastatic adenocarcinomas, in addition to selecting adenocarcinoma markers (MOC31, BerEP4, BG8, B72.3, CEA), organ‐specific markers such as estrogen receptor (ER), progesterone receptor (PR), GCDFP15, and mammaglobin for breast cancer, PAX8, PAX2, RCC, CD15 for renal cancer, and PAX8, ER, PR for ovarian cancer should also be included. It should be noted that most epithelioid mesotheliomas express GATA3, and some may also be positive for PAX8. Immunohistochemical detection of BAP1 and EZH2 is a reliable marker for differentiating benign mesothelial proliferation from malignant mesothelioma.^35^, ^36^, ^37^ Immunohistochemical detection of MTAP can be used as a substitute immunohistochemical marker for homozygous deletion of *CDKN2A*.^36^, ^38^ Immunohistochemical testing of TOP2A expression levels is associated with the prognosis of MPM. It has been found that the TOP2A positivity rate in tumor tissue is an independent prognostic factor and is correlated with high expression and good prognosis.^39^

### Molecular diagnosis

The common mutated genes for MPM are *BAP1, CDKN2A* and *NF2*.^40^ *BAP1* is the most common mutated gene in MPM. *BAP1* Germline mutations are associated with other malignant tumors such as uveal melanoma and renal cell carcinoma, collectively known as *BAP1* tumor predisposition syndrome. *BAP1* mutations can be detected in 45%–100% in MPM, and are mainly present in epithelioid mesothelioma, which is associated with favorable prognosis. For patients with peritoneal mesothelioma, no asbestos exposure, younger age, and previous tumors history, *BAP1* gene testing is recommended.^41^, ^42^ *CDKN2A* mutation is associated with poor prognosis, and the positivity rate of *CDKN2A* in sarcomatoid mesothelioma is almost 100%.^43^ About 50% of patients have *NF2* heterozygous or homozygous deletion mutations.^40^ *BAP1* and *CDKN2A* gene mutations do not have a 100% specificity for the diagnosis of malignant mesothelioma, but they can help distinguish MPM from benign pleural diseases. Studies have found that *KRAS* occurs in 13.7% of MPM cases and is associated with shortened median survival.^44^

## SURGICAL TREATMENT

**Consensus 6:** Patients with epithelioid mesothelioma in stages I–IIIA are good candidates for surgical treatment. Therefore, for patients with operable epithelioid mesothelioma, the expert group recommends surgical treatment, using either pleurectomy or decortication as the surgical approach **(strongly recommended).**

The goal of surgical treatment for MPM is to remove all visible or palpable tumors, which is called complete cytoreduction surgery. If the tumor cannot be completely removed due to chest wall invasion in multiple areas, the surgery should be stopped. For patients with stage I–IIIA MPM, there is a possibility of surgical resection, which can be evaluated after multidisciplinary team discussion (See Appendix – Staging (Supplementary Table 1)).^45^, ^46^, ^47^, ^48^, ^49^ For stage IIIB–IV MPM, surgery is not recommended. Although studies have shown that patients with stage I–II sarcomatoid mesothelioma may benefit from surgery with extended overall survival (OS), the perioperative complications and mortality rate are significantly higher than those of nonsarcomatoid mesothelioma patients. Therefore, surgery is also not recommended.^50^ Generally, tumor reduction surgery is not recommended unless it is safe to remove most of the tumor, reduce the tumor burden, and facilitate postoperative treatment.^51^, ^52^ The main surgical methods for the resection of MPM include: (1) pleurectomy/decortication (P/D), which completely removes the affected pleura and all tumor tissue; (2) extrapleural pneumonectomy (EPP), which involves the extensive resection of the affected pleura, lung, ipsilateral diaphragm, and pericardium.^53^ Both P/D and EPP aim to remove tumors that are visible or palpable to the naked eye. At least three groups or more of mediastinal lymph nodes should be removed, but it is difficult to achieve R_0_ resection in both.^54^, ^55^ Due to the lack of results from large randomized controlled clinical trials, there is controversy over the choice of surgical procedures for MPM. In patients with stage II–IIIA MPM, EPP surgery is often performed to remove visible tumors.^50^ However, EPP surgery has a wider range of resection, but there is a higher incidence of perioperative complications and mortality, and there is controversy over its effectiveness in improving patient prognosis.^48^ A multicenter retrospective analysis (*n* = 663) showed that the overall survival of patients who underwent P/D was better than that of patients who underwent EPP.^56^ The meta‐analysis shows that the perioperative mortality rate (within 30 days) of P/D is lower than that of EPP, while in terms of long‐term mortality rate (2 years), the two are similar.^46^, ^57^ In clinical practice, the surgical approach should be carefully selected by an experienced multidisciplinary team based on factors such as the histological subtype, location, staging, lung reserve, surgical experience, and feasibility of adjuvant and intraoperative treatment strategies for the tumor (See Appendix – Comprehensive Treatment Principles).

## RADIOTHERAPY AND OTHER PHYSICAL THERAPY

**Consensus 7:** For postoperative patients with good PS score, lung function, and kidney function, and there is no lesion in the abdomen, contralateral chest or other parts, postoperative half‐side thoracic adjuvant radiotherapy can be considered to reduce local recurrence rate and prolong survival. Patients who require oxygen therapy after surgery cannot improve survival with adjuvant radiotherapy and should not consider adjuvant radiotherapy **(strongly recommended).**

**Consensus 8:** Tumor treating fields (TTFields) therapy is convenient, widely applicable, and has minimal side effects. In institutions where conditions permit, it can be used as a new mode of comprehensive treatment **(recommended).**

### Radiotherapy

Radiotherapy can have a positive therapeutic effect on MPM.^58^ Since 2000, the application of highly conformal radiotherapy techniques, such as intensity‐modulated radiotherapy (IMRT), has allowed researchers to optimize high‐dose radiotherapy for the entire hemithorax. However, radiotherapy is generally not recommended as a standalone treatment and should be used as part of a multidisciplinary approach. Radiotherapy can be used as palliative therapy to relieve chest pain, alleviate bronchial or esophageal obstruction, and treat other symptoms related to MPM, such as brain or bone metastases. The optimal timing for radiotherapy should be determined through discussion among the multidisciplinary team. Prospective single‐arm trials have shown that completion of high‐dose hemithoracic radiotherapy after EPP surgery can result in a median survival time of 23.0–39.4 months, independent of chemotherapy response, suggesting that IMRT can benefit EPP patients.^57^ After EPP surgery, adjuvant radiotherapy may reduce the local recurrence rate.^59^, ^60^, ^61^, ^62^ If the patient has a good PS score, lung function, and kidney function, radiotherapy can be performed. In patients who cannot undergo surgical treatment or have incomplete surgical resection, conventional half‐chest high‐dose radiotherapy cannot improve survival rate and is accompanied by significant adverse reactions.^63^ A phase II clinical trial (IMPRINT) evaluated the safety of half thoracic intensity‐modulated radiation therapy (IMRT) after induction chemotherapy and surgery in patients with malignant pleural mesothelioma (MPM) (*n* = 27). The results showed that the incidence of radiation pneumonitis was 30% (95% CI: 14%–50%). In operable patients, the 2‐year overall survival rate was 59%. 22% (6/27) of patients had mediastinal lymph node metastasis, and 48% (13/27) of patients had distant metastasis.^64^ According to the results of this study,^63^ in centers with rich experience in radiotherapy, half‐sided thoracic IMRT can be considered for some patients with MPM who have undergone certain induction chemotherapy and P/D surgery. There has been controversy over whether postoperative radiotherapy (prophylactic radiotherapy) can be used to prevent recurrence along the surgical path after pleural surgery. A clinical trial from France showed that radiotherapy can prevent postoperative recurrence, but other clinical trials did not show benefits.^65^, ^66^, ^67^ In a phase III randomized trial (SMART trial), researchers compared the postoperative recurrence rate between prophylactic radiotherapy and delayed radiotherapy, where the delayed radiotherapy group only received radiotherapy when surgical path transfer occurred. The results showed no difference in the recurrence rate between the prophylactic radiotherapy group and the delayed radiotherapy group (9% [9/102] and 16% [16/101], OR = 0.51, 95% CI: 0.16–1.32); prophylactic radiotherapy did not improve quality of life or reduce chest pain or the need for painkillers. However, if patients did not receive postoperative chemotherapy, prophylactic radiotherapy could reduce the risk of surgical path transfer (OR = 0.16, 95% CI: 0.02–0.93, *p* = 0.021).^68^ The recommended radiotherapy dose for different treatment purposes is shown in Table 2 (See Appendix – Comprehensive Treatment Principles).

**TABLE 2:** Recommended radiotherapy doses for malignant pleural mesothelioma patients according to different treatment objectives.

Treatment type and timing	Radiation therapy dosage and cycle
EPP postoperative adjuvant radiotherapy	45–60 Gy, 1.8–2 Gy per session, for a total of 5–6 weeks
P/D postoperative adjuvant IMRT radiotherapy	45–60 Gy, 1.8–2 Gy per session, for a total of 5–6 weeks
Palliative treatment: Pain caused by chest wall nodules	20–40 Gy, ≥4 Gy per session, with a treatment time of 1–2 weeks, or 20–40 Gy, 3 Gy per session, with a treatment time of 2 weeks
Multiple brain or bone metastases	30 Gy, 3 Gy per session, with a treatment time of 2 weeks

Abbreviations: EPP, extrapleural pneumonectomy; IMRT, intensity‐modulated radiation therapy; P/D, pleurectomy/decortication; PS, performance status.

### Other physical therapy

Tumor treating fields (TTFields) is a portable, noninvasive local antitumor treatment method. By attaching a disposable sensor to the chest, a low‐intensity (1–3 V/cm), intermediate‐frequency (100–300 kHz), alternating electric field is generated that acts on the microtubules of proliferating cancer cells in two directions, interfering with tumor cell mitosis, causing affected cancer cells to undergo apoptosis, and thus inhibiting tumor growth. In the STELLAR study, patients with unresectable MPM received electric field therapy plus pemetrexed and platinum‐based chemotherapy. The results showed that the disease control rate (DCR) reached 97%, the objective response rate (ORR) was 40%, and the median survival time was 18.2 months. The median survival time for patients with epithelioid mesothelioma could be extended to 21.2 months. The median survival time for patients with nonepithelioid mesothelioma was 12.1 months. In May 2019, TTFields was approved by the US FDA for comprehensive treatment. The common grade 1–2 adverse events of TTFields are skin reactions under the sensor, which can be treated with local corticosteroids or temporary interruption of treatment, and the skin reactions disappear, suggesting that it can be used as a new model for future comprehensive treatment.^69^

## MEDICAL TREATMENT

**Consensus 9:** For stage IIIB–IV and unresectable stage I–IIIA patients, first‐line treatment is recommended with pemetrexed combined with platinum‐based drugs and bevacizumab or with nivolumab combined with ipilimumab; for sarcoma type, nivolumab combined with ipilimumab is preferred **(strongly recommended).**

**Consensus 10:** For patients who have not received immunotherapy in the first‐line treatment, nivolumab + ipilimumab, nivolumab, and pembrolizumab monotherapy are also recommended as second‐line and subsequent treatment options **(strongly recommended).**

**Consensus 11:** For patients who have not used pemetrexed in the first‐line treatment, pemetrexed can be used as second‐line option **(strongly recommended).**

### Systemic chemotherapy

#### First‐line chemotherapy

Chemotherapy can be used for stage IIIB–IV and unresectable stage I–IIIA patients. The first‐line treatment for MPM is a combination of pemetrexed and cisplatin or a triple combination of pemetrexed, cisplatin, and bevacizumab. Carboplatin is recommended for patients who are intolerant to cisplatin. In a phase III randomized trial, researchers compared the efficacy of pemetrexed and cisplatin with single‐agent cisplatin in MPM patients who were not suitable for surgery. The results showed that combination therapy extended the median overall survival (OS) by 2.8 months (12.1 vs. 9.3 months, *p* = 0.02) compared to single‐agent cisplatin.^70^ This study established the cornerstone position of pemetrexed and cisplatin combination therapy in MPM chemotherapy. In a multicenter phase III randomized trial (IFCT‐GFPC‐0701MAPS), researchers compared the efficacy and safety of pemetrexed, cisplatin, and bevacizumab with pemetrexed and cisplatin in the treatment of unresectable MPM. The results showed that combination therapy with bevacizumab extended the median OS by 2.7 months (18.8 vs. 16.1 months, HR = 0.77, *p* = 0.0167) compared to chemotherapy alone.^71^ This study established the first‐line treatment position of pemetrexed, cisplatin, and bevacizumab. Several phase III clinical trials have shown that pemetrexed and carboplatin can also achieve good survival time.^72^, ^73^, ^74^ An expanded access trial included 1704 patients with unresectable MPM, and the results showed that the median PFS and OS of patients treated with pemetrexed and cisplatin or pemetrexed and carboplatin were similar.^75^ For patients with poor performance status (PS) scores unable to tolerate cisplatin treatment, pemetrexed and carboplatin chemotherapy can be used. In a phase II clinical trial, researchers evaluated the efficacy of pemetrexed, carboplatin, and bevacizumab in the treatment of unresectable MPM (*n* = 76). The median OS was 15.3 months, the objective response rate (ORR) was 34%, and the disease control rate (DCR) was 58%.^76^ The results of a phase III clinical trial showed that the median OS of patients treated with gemcitabine and cisplatin was 9.6–11.2 months.^77^, ^78^, ^79^ This alternative treatment option can be used for patients who are intolerant to pemetrexed treatment. A multicenter randomized controlled study (MS01) showed that single‐agent vinorelbine can also be used to treat patients who are intolerant to platinum‐based drugs.^78^

#### Second‐line chemotherapy

For patients who have not received pemetrexed as first‐line treatment, second‐line treatment is recommended.^80^ For patients who have received pemetrexed in first‐line treatment with treatment failure, pemetrexed can still be used again, especially for young patients with good PS scores and long progression‐free survival after first‐line treatment.^81^ A meta‐analysis included three phase III studies, 18 phase II studies, and eight retrospective studies, all exploring second‐line or subsequent treatment options. None of the three phase III studies showed an overall survival benefit, while retrospective studies showed some benefit with gemcitabine and vinorelbine, which can be used when no other options are available.^82^ A phase II trial called RAMES, a multicenter, randomized, double‐blind, placebo‐controlled study, aimed to evaluate the efficacy and safety of gemcitabine combined with VEGFR‐2 antibody ramucirumab in second‐line treatment of malignant pleural mesothelioma (MPM) (*n* = 161). The results showed that the median overall survival of patients in the gemcitabine plus ramucirumab group was significantly prolonged (13.8 vs. 7.5 months, HR: 0.71, 70% CI: 0.59–0.85; *p* = 0.028). The most common treatment‐related grade 3–4 adverse events were neutropenia and hypertension, and there were no treatment‐related deaths, indicating good safety.^83^ An international, multicenter, randomized, open‐label phase II trial called ARCS‐M aimed to evaluate the efficacy of the antibody‐drug conjugate anetumab ravtansine (an all‐human anti‐mesothelin antibody and toxic group DM4) compared with vinorelbine in second‐line treatment of MPM patients. The study results showed that 105 patients (63%) in the anetumab ravtansine group and 43 patients (52%) in the vinorelbine group experienced disease progression or death, and the median progression‐free survival was 4.3 and 4.5 months, respectively (HR = 1.22, *p* = 0.86). The most common grade 3 or higher adverse reactions were neutropenia (1% vs. 39%), pneumonia (4% vs. 7%), neutrophil count reduction (1% vs. 17%), and dyspnea (6% vs. 4%). Twelve patients (7%) in the anetumab ravtansine group and 11 patients (15%) in the vinorelbine group experienced serious treatment‐related adverse reactions. In addition, 10 patients in the anetumab ravtansine group died from treatment‐related adverse reactions. Therefore, although anetumab ravtansine showed controllable safety in patients with malignant pleural mesothelioma, it was not superior to vinorelbine.^84^

### Immunotherapy

#### First‐line immunotherapy

CheckMate‐743 is an open‐label, multicenter, randomized phase III clinical trial aimed at evaluating the efficacy of nivolumab plus ipilimumab compared to standard chemotherapy for untreated MPM. The results showed that nivolumab plus ipilimumab significantly reduced the risk of death in patients with unresectable MPM by 26% compared to standard chemotherapy (pemetrexed plus cisplatin or carboplatin). The median overall survival (OS) for the nivolumab plus ipilimumab group was 18.1 months, which was superior to the chemotherapy group's 14.1 months (HR = 0.74, 96.6% CI: 0.60–0.91, *p* = 0.0020). Subgroup analysis showed that patients with nonepithelioid MPM and programmed cell death‐ligand 1 (PD‐L1) expression ≥1% (HR = 0.69) benefited more in terms of OS.^85^ The CheckMate‐743 study confirmed for the first time that first‐line treatment with dual immunotherapy can improve the survival of patients with unresectable MPM. Patients with sarcomatoid MPM have a worse prognosis and poor response to chemotherapy, but they can benefit more from the combination of nivolumab plus ipilimumab. Therefore, this dual immunotherapy regimen is expected to become the standard first‐line treatment for MPM, especially nonepithelioid mesothelioma. In addition, the CheckMate‐743 study showed that PD‐L1 expression may be a predictive factor for the effectiveness of dual immunotherapy. In terms of safety, the dual immunotherapy regimen had a lower incidence of adverse events than the chemotherapy group, with treatment‐related adverse events (TRAE) occurring in 79% and 82% of the dual immunotherapy and chemotherapy groups, respectively, and grade 3–4 TRAE occurring in 30% and 32%, respectively.^85^ The US Food and Drug Administration (FDA) approved the use of nivolumab plus ipilimumab dual immunotherapy for first‐line treatment of MPM in 2010. In June 2021, this indication was approved in China. The DREAM study is the first attempt to use the PD‐L1 inhibitor durvalumab in combination with platinum‐containing chemotherapy as first‐line treatment for MPM. It is a multicenter, single‐arm, phase II study that enrolled 54 adult MPM patients with various pathological subtypes who were untreated. The patients were treated with durvalumab plus pemetrexed plus cisplatin and received durvalumab maintenance (up to 12 months). The primary endpoint of the study was a 6‐month progression‐free survival rate of 57%, and the partial response rate was 48%. Compared to chemotherapy alone, chemotherapy plus immunotherapy improved patients' 6‐month progression‐free survival rate and ORR, and adverse reactions were tolerable.^86^

#### Second‐line and subsequent immunotherapy

The exploration of immunotherapy in MPM was first carried out in second‐line treatment. IFCT‐1501MAPS2 is a multicenter, randomized, noncontrolled phase II study (*n* = 125) that evaluated the efficacy of nivolumab ± ipilimumab as second‐line treatment for MPM. The results showed that the median OS of patients treated with nivolumab + ipilimumab was 15.9 months (95% CI: 10.7 months to not reached), and the 1‐year survival rate was 58%. The median OS of patients treated with nivolumab monotherapy was 11.9 months (95% CI: 6.7–17.7 months), and the 1‐year survival rate was 49%. PD‐L1 high expression was positively correlated with overall response rate, especially when PD‐L1 expression was ≥25%. Dual immunotherapy can improve efficacy, but also increases the incidence of adverse reactions. The incidence of grade 3–4 adverse events in the combination therapy group was significantly higher than that in the monotherapy group (26% vs. 14%).^87^ The INITIATE study is a single‐arm, phase II clinical trial of nivolumab + ipilimumab for the treatment of recurrent MPM. The study showed that after 12 weeks of treatment, the disease control rate (DCR) was 68% (23/34), with partial response achieved in 29% (10/34) of patients and disease stabilization in 38% (13/34) of patients. In total, 94% of patients experienced treatment‐related adverse reactions, with a 34% incidence of grade 3 adverse reactions.^88^ In the 2017 KEYNOTE‐028 study, the results of first‐line treatment with pembrolizumab monotherapy for MPM were reported for the first time. A total of 25 patients who met the inclusion criteria were enrolled, all of whom were PD‐L1 positive, including 18 with epithelioid mesothelioma, four with sarcomatoid or biphasic mesothelioma, and three with undetermined histological type of mesothelioma. A total of 15 patients had received first‐line treatment, and eight had received second‐line treatment. A total of 22 patients (88%) had received platinum‐containing treatment. The study showed that five patients achieved partial response, with a DCR of 72% (18/25), and the median OS and median PFS of patients were 18 and 5.4 months, respectively, with a 1‐year survival rate of 62.6%.^89^ Several small sample studies of immunotherapy checkpoint inhibitors as monotherapy for MPM were conducted during the same period, including the Merit, NivoMes, JAVELIN, and UChicago studies. The immunotherapy checkpoint inhibitors used as monotherapy included nivolumab (used in both the Merit and NivoMes studies), avelumab, and pembrolizumab. The results showed that the ORR of patients was 29, 24, 8, and 19%, respectively, and the DCR was 68, 47, 58, and 66%, respectively. The median OS was 17.3, 11.8, 10.7, and 11.5 months, respectively,^90^, ^91^, ^92^ which did not surpass the KEYNOTE‐028 study. The CONFIRM study was the first phase III clinical study to compare anti‐PD‐1 monoclonal antibodies with placebo in patients with recurrent malignant mesothelioma (95% of whom had malignant pleural mesothelioma). The study included adult patients with confirmed metastatic malignant mesothelioma who had received at least one prior treatment and were not candidates for surgical resection. Patients were randomly assigned to receive nivolumab (*n* = 221) or placebo (*n* = 111) at a 2:1 ratio. The primary endpoints were OS and PFS. The results showed that nivolumab treatment increased OS, with a median OS of 9.2 (95% CI: 7.5–10.8) and 6.6 months (95% CI: 5.0–7.5) in the nivolumab and placebo groups, respectively (HR = 0.72, 95% CI: 0.55–0.94, *p* = 0.018).^90^ The 1‐year OS rate was 39.5% in the nivolumab group and 26.9% in the placebo group. The PFS data also showed that nivolumab was significantly superior to placebo, with a median PFS of 3.0 and 1.8 months (HR = 0.61, 95% CI: 0.48–0.77, *p* < 0.001), and the 1‐year PFS rates were 14.5 and 4.9% in the two groups, respectively. In this study, the epithelioid subtype showed more benefit, with a median OS of 9.4 and 6.6 months (HR = 0.71, 95% CI: 0.53–0.95, *p* = 0.021) and a 1‐year overall survival rate of 40.0 and 26.7% in the study and control groups, respectively.^93^

#### Combined immunotherapy

Vascular endothelial growth factor (VEGF), such as angiogenesis factor, induces immune suppression in the tumor microenvironment (TME) by causing vascular abnormalization, inhibiting antigen presentation and immune effector cell function, or enhancing regulatory T cells, myeloid‐derived suppressor cells, and tumor‐associated macrophage immune suppression effects.^94^ Immune suppressor cells can also promote tumor angiogenesis, leading to a series of inhibitory immune effects.^95^ The VEGF‐mediated immune suppression in the TME and the adverse effects on the efficacy of PD‐1/PD‐L1 inhibitors suggest that the combination of PD‐1/PD‐L1 antibodies and anti‐VEGF drugs may synergistically improve the tumor microenvironment and normalize it. Currently, this combination therapy has been carried out in various tumors, including non‐small cell lung cancer, renal cell carcinoma, hepatocellular carcinoma, and more similar studies are underway, with the hope of achieving better efficacy and further improving the current status of immunotherapy.^96^, ^97^ In particular, the combination of anlotinib and ICI therapy has been proven to be safe and significantly improve efficacy in case reports and clinical studies,^98^, ^99^ and in basic research, anlotinib has been shown to improve the efficacy of immunotherapy by adjusting the tumor microenvironment.^100^, ^101^

Most chemotherapy drugs act directly on cells and their impact on the immune system has not been considered. However, the interaction between chemotherapy and immunotherapy has been formally demonstrated in mouse models, and intact mice with an immune system have shown significantly improved tumor responses to anthracycline drugs. So far, multiple studies have shown the contribution of cytotoxic chemotherapy to anticancer immunity, and some FDA and/or NMPA‐approved immunotherapies have been combined with chemotherapy. Clinical trials have been conducted in patients with MPM to explore the safety and efficacy of immune combined chemotherapy.

In future, the combination of immunotherapy with bevacizumab or multitargeted small molecule antitumor angiogenesis drugs and chemotherapy is expected to improve the efficacy of first‐ and second‐line treatments for MPM patients, providing them with a more hopeful treatment option.^102^

### Targeted therapy

Genomic studies have shown that there are no clear driver gene mutations detected in the tumor tissue of MPM patients, while the inactivation of tumor suppressor genes, including *CDKN2A/2B*, *BAP1*, *NF2*, *LAST1/2*, is dominant. Correcting the inactivated tumor suppressor genes is much more difficult than targeting tumor driver genes and previous targeted therapy studies for MPM have mostly ended in failure.^103^, ^104^, ^105^ Relevant studies on targeted therapy for downstream or related genes of these abnormal genes are currently underway. *CDKN2A* encodes P16, which negatively regulates *CDK4/6*, thereby inhibiting tumor cell proliferation. Studies have shown that *CDK4/6* inhibitors may be effective in treating MPM patients with *CDKN2A* mutations that cause *CDK4/6* upregulation.^64^, ^106^ A phase II clinical trial called SIGNATURE included MPM patients with *CDKN2A* gene mutations resulting in functional loss, who were given CDK4/6 inhibitor LEE011. The results showed that among the 105 analyzed patients, the proportion of CR/PR/SD/NE patients was 2.9, 15.2, 67.6, and 14.3%, respectively (0 cases, 3 cases, 16 cases, 71 cases, and 15 cases) (NCT02187783). Another clinical trial using CDK4/6 inhibitors for MPM patients with *CDKN2A* gene mutations resulting in functional loss is currently underway (NCT03654833). *BAP1* mutations frequently occur in MPM patients, leading to DNA double‐strand damage repair defects, causing genomic instability and thus the occurrence and development of the disease. PARP is a synthetic lethal target in the homologous recombination repair pathway, and its inhibitors are effective against various tumors with BAP1 deficiency.^107^, ^108^ Studies have shown that MPM cells with different *BAP1* mutation statuses show no significant difference in sensitivity to PARP inhibitors.^109^ In in vivo and in vitro experiments, MPM with *BAP1* mutations is sensitive to Zeste homolog 2 (*EZH2*). The US Food and Drug Administration (FDA) has approved the world's first EZH2 inhibitor tazemetostat for marketing, with the approved indication being for metastatic or advanced epithelioid sarcoma that is not suitable for surgical resection. Currently, a phase II clinical study in relapsed MPM shows that tazemetostat has good antitumor effects and is relatively safe for patients with BAP1 gene mutations (NCT02860286). In melanoma, patients with BAP1 gene mutations are sensitive to targeted HDAC4 therapy. However, an international multicenter randomized clinical trial showed that HDAC4 inhibitor vorinostat used for second‐ or third‐line treatment of advanced MPM patients did not improve survival and is not recommended (NCT00128102).^110^ A clinical case report showed that a MPM patient with *CD74‐ROS1* fusion detected by next‐generation sequencing (NGS) large panel achieved persistent complete remission after treatment with crizotinib.^111^

### Other treatments

Oncolytic virus is a novel antitumor treatment strategy, especially for MPM, which shows promising application prospects due to the operability of intrapleural injection.^112^ Studies have shown that oncolytic virus therapy is more effective for patients with type I interferon homozygous deficiency.^113^ A phase I–II clinical trial using oncolytic herpes virus to treat MPM showed antitumor effects and good safety.^114^ Targeted mesothelin therapy is also one of the exploration directions in the field of MPM treatment, including immunotoxins and CAR‐T cell therapy. In the evaluation of the safety and optimal tolerated dose of immunotoxin LMB‐100 in a phase I clinical study, patients received PD‐1 inhibitors for maintenance therapy after LMB‐100 treatment; the results showed partial remission in three cases and complete remission in one case, with the latter achieving a disease‐free survival of 37 months.^115^ The principle of CAR‐T cell therapy is to modify T cells in vitro to express receptor fragments that specifically recognize tumor surface antigens, and then input the T cells that recognize tumor antigens into the patient's body to achieve direct targeting of cancer cells and exert immune killing effects. The safety and efficacy of CAR‐T cell therapy in advanced MPM are in phase I trials. The results of a phase I clinical study conducted by Memorial Sloan‐Kettering Cancer Center showed that in 27 patients (25 of whom were MPM patients) CAR‐T treatment was safe and well‐tolerated, with CAR‐T cells detected in the peripheral blood for more than 100 days in 39% of patients, and 18 patients also safely received pembrolizumab treatment, with a median overall survival of 23.9 months and a 1‐year overall survival rate of 83%.^116^

In summary, the principles and recommended schemes for internal medicine treatment of MPM are shown in Table 3 (See Appendix – Assessment of Response to Treatment in Malignant Pleural Mesothelioma – Modified Response Evaluation Criteria in Solid Tumors 1.1 [mRECIST v1.1]; Appendix – Medical treatment regimens [Supplementary Table 2; Supplementary Table 3; Supplementary Table 4]; Appendix – Comprehensive Treatment Principles).

**TABLE 3:** Principles and plans for internal treatment of malignant pleural mesothelioma.

Treatment lines	Class I evidence	Class II evidence
First‐line treatment	Pemetrexed + cisplatin	Pemetrexed + carboplatin ± bevacizumab
	Pemetrexed + cisplatin + bevacizumab	Durvalumab + pemetrexed + cisplatin
	Nivolumab + ipilimumab	Gemcitabine + cisplatin
		Pemetrexed
		Vinorelbine
Second‐line or subsequent treatment	Pemetrexed	Vinorelbine
		Gemcitabine
		Pembrolizumab
		Nivolumab ± ipilimumab

## PALLIATIVE AND SUPPORTIVE TREATMENTS

**Consensus 12:** The focus of palliative and supportive therapy for malignant pleural mesothelioma is pain management and relief of respiratory distress and hypoxia symptoms. For patients who cannot undergo radical resection, drug therapy is the preferred method for cancer pain management. Substantial tumor reduction surgery can be preserved. If pleural effusion needs to be treated, it is recommended to use talc pleurodesis or pleural catheter drainage **(strongly recommended).**

Palliative and supportive therapy is an important part of the cancer prevention and control system, focusing on relieving pain, other severe disease symptoms, and emotional distress, and improving quality of life for patients. For patients with MPM, drug therapy is the preferred method for cancer pain management. For moderate pain, weak opioid drugs such as codeine or tramadol can be added, and for severe pain, strong opioid drugs can be added.^117^ Malignant pleural mesothelioma patients exhibit two of the most difficult types of pain to control: cancer‐induced bone pain and neuropathic pain. Due to the local effects on nerve vascular bundles, the pain experience of MPM patients has a strong neuropathic component.^117^ In addition to opioid drugs, auxiliary drugs targeting specific neuropathic pain mechanisms can also be used, with the most commonly used being tricyclic antidepressants and antiepileptic drugs such as gabapentin and pregabalin.^117^, ^118^

Malignant pleural mesothelioma patients can undergo palliative surgery. The appropriate population for palliative surgery includes patients who are not suitable for complete resection due to tumor staging or disease status. For MPM patients with obvious pain and mediastinal syndrome, palliative radiotherapy is usually performed.

Clinically, MPM patients often present with chest pain, dyspnea, weight loss, and pleural effusion. Therefore, when performing palliative and supportive therapy, attention should be paid to the treatment of chest pain, dyspnea, and pleural effusion, as well as nutritional support. Adverse reactions to radiotherapy, chemotherapy, targeted drugs, and immune checkpoint inhibitors should also be addressed, including radiation‐induced xerostomia, radiation pneumonitis, radiation enteritis, radiation dermatitis, cardiac toxicity, bone marrow suppression, liver damage, pulmonary interstitial fibrosis, skin toxicity, neuropathy, immune pneumonia, muscle and joint pain, etc.^119^ Corresponding drug therapy and clinical management should be provided.

## FOLLOW‐UP

**Consensus 13:** Malignant pleural mesothelioma patients should undergo chest and/or abdominal CT re‐examination every 3–6 months after active treatment **(strongly recommended).**

Currently, there is a lack of high‐level evidence‐based medicine for the follow‐up mode of MPM. Considering the poor prognosis of MPM, regular follow‐up can detect tumor progression early. In July 2015, the European Society for Medical Oncology released clinical diagnosis and treatment guidelines for MPM, which suggested that when patients have clinical symptoms, CT should be used for efficacy evaluation; the follow‐up time should depend on the local recommendations for patient treatment or the treatment plan specified in the study.^21^ In 2020, the European Respiratory Society, European Society of Thoracic Surgeons, European Association for Cardio‐Thoracic Surgery, and European Society for Radiotherapy and Oncology jointly released management guidelines for MPM. For patients receiving chemotherapy, chest CT re‐examination should be performed when symptoms such as dyspnea and chest pain occur. Currently, PET‐CT and MRI are not recommended as routine follow‐up examinations, and there is insufficient evidence to support the use of biomarkers for MPM follow‐up. Monitoring disease progression should be guided by signs and symptoms that occur during clinical follow‐up. Overall, MPM patients should undergo chest and/or abdominal CT re‐examination every 3–6 months after active treatment.^120^

## AUTHOR CONTRIBUTIONS

Anwen Liu, Wenzhao Zhong and Wenfeng Fang participated in the design of the expert consensus. Qian Wang, Chunwei Xu, Wenxian Wang and Yongchang Zhang conceived of the expert consensus, and participated in its design and other authors coordination and helped to draft the expert consensus. All authors read and approved the final manuscript.

## CONFLICT OF INTEREST STATEMENT

There are no conflicts of interest to disclose.

## Supporting information


Appendix S1.
