# Supplementary material for: Chinese expert consensus on the diagnosis and treatment of malignant pleural mesothelioma
Source: Thorac Cancer. 2023 Jul 17;14(26):2715–31. doi: 10.1111/1759-7714.15022 (PMC10493492; doi:10.1111/1759-7714.15022)
Supplement: Supplementary file 1 — Appendix S1. [file TCA-14-2715-s001.docx]

**Appendix - Staging**

On January 1, 2018, the eighth edition of the cancer staging system developed by the American Joint Committee on Cancer (AJCC) was implemented globally, which includes the TNM staging for malignant pleural mesothelioma (MPM) as shown in Supplementary Table 1 of the appendix.

Supplementary Table 1: Corresponding table of TNM staging for malignant pleural mesothelioma in the 8th edition of the American Joint Committee on Cancer.

| TNM stage | Primary tumor (T) | Regional lymph nodes (N) | Distant metastasis (M) |
| --- | --- | --- | --- |
| StageⅠA | T1 | N0 | M0 |
| StageⅠB | T2-3 | N0 | M0 |
| StageⅡ | T1-2 | N1 | M0 |
| Stage ⅢA | T3 | N1 | M0 |
| Stage ⅢB | T1-3 | N2 | M0 |
|  | T4 | Any N | M0 |
| StageⅣ | Any T | Any N | M1 |

(1) Primary tumor (T)

T_X_: Primary tumor cannot be evaluated;

T0: No evidence of primary tumor;

T1: Primary tumor is limited to the ipsilateral pleural wall, with or without involvement of the visceral pleura, mediastinal pleura, or diaphragmatic pleura;

T2: Tumor involves the ipsilateral pleural surface of one site (parietal pleura, mediastinal pleura, diaphragmatic pleura, visceral pleura), and has at least one of the following characteristics: invasion of the diaphragm; invasion of lung parenchyma through the visceral pleura;

T3: Locally advanced but potentially resectable tumor. The tumor involves the pleural surfaces of the ipsilateral side (parietal pleura, mediastinal pleura, diaphragmatic pleura, visceral pleura), and has at least one of the following characteristics: invasion of the parietal pleura; invasion of mediastinal fat; a single, completely resectable tumor lesion invading soft tissues of the chest wall; non-transmural invasion of the pericardium;

T4: Locally advanced technically unresectable tumor. The tumor involves the pleural surfaces of the ipsilateral side (parietal pleura, mediastinal pleura, diaphragmatic pleura, visceral pleura), and has at least one of the following characteristics: diffuse infiltration or multiple lesions of the chest wall, with or without rib destruction; direct invasion into the abdominal cavity through the diaphragm; direct invasion of the contralateral pleura; direct invasion of mediastinal organs; direct invasion of the spine; penetration of the inner surface of the pericardium, with or without pericardial effusion, or invasion of the myocardium.

(2) Regional lymph nodes (N)

Nx: unable to evaluate lymph node metastasis;

N0: No regional lymph node metastasis;

N1: Metastasis of lymph nodes on the same side as the bronchus, lung, hilum, or mediastinum (including ipsilateral internal mammary, peridiaphragmatic, pericardial fat pad, and intercostal lymph nodes);

N2: contralateral mediastinum, ipsilateral or contralateral supraclavicular lymph node metastasis.

(3) Distant metastasis (M)

M0: no distant metastasis;

M1: distant metastasis.

**Appendix - Assessment of Response to Treatment in Malignant Pleural Mesothelioma - Modified Response Evaluation Criteria in Solid Tumors 1.1 (mRECIST v1.1) ^1,2^.**

Malignant pleural mesothelioma (MPM) is typically characterized by irregular pleural thickening or pleural masses on CT imaging, with a "peel-like" growth pattern being a common feature. It often affects one side of the pleura, but can occasionally be seen on both sides. This unique growth pattern limits the application of the Response Evaluation Criteria in Solid Tumours (RECIST) in clinical trials of MPM. To address this issue, a study in 2004 proposed using "thickness" instead of "longest diameter" to represent the tumor burden of MPM and validated this measurement method. Subsequently, in 2018, this assessment method was refined and revised to form the mRECIST 1.1 criteria, which are applicable to MPM.

**1 Summary of mRECIST1.1**

**Definition**

(1) mRECIST1.1 defines the concept of "measuring site" for the evaluation of mesothelioma tumor imaging. The tumor thickness measured from the measuring site forms the basis for the evaluation of mesothelioma tumor remission.

(2) mRECIST1.1 suggests lowering the definition of the minimum measurable disease for mesothelioma from 10mm to 7mm.

**Measurement requirements**

(3) mRECIST1.1 stipulates that up to 6 pleural measuring sites can be selected at baseline (no more than 2 sites per CT section, no more than 3 selected sections, and at least 1cm apart for each section); each site must meet the minimum measurable disease standard.

(4) If the baseline scan measurement line segment is viewed during subsequent scan measurements, the preferred association between the measuring site and the anatomical landmark can be relaxed. Sites that are "measurable and repeatable" should continue to be considered.

(5) Only the measuring sites selected at baseline will be included in the total tumor measurement of subsequent CT scans.

(6) Measurement should preferably be performed on the soft tissue window of the CT cross-section.

(7) Measuring sites located above the left atrium and below the aortic arch are preferred.

(8) The measurement of tumor thickness should not include pleural effusion.

(9) If there are lesions on both sides, the pleura of the two halves of the chest is considered as one "organ", so that up to 6 pleural measuring sites are distributed in the two halves of the chest; although no more than 2 sites can be selected in any CT section of each half of the chest, the section selection of each half of the chest should be considered independently.

**Measurement process**

(10) mRECIST1.1 recommends that the same observer use the same image display parameters (such as contrast and brightness settings) to measure all CT scans of a patient.

(11) mRECIST1.1 recommends storing images of the baseline scan measurement line segment for visual reference during subsequent scan measurements.

(12) After obtaining the baseline tumor thickness measurement value at a measuring site, the measurement direction of all subsequent time points at that site should be consistent with the baseline scan measurement direction.

(13) If the relationship between the chest wall and mediastinum changes, making it impossible to observe the two measuring sites of the baseline scan section on the same section of subsequent scans, the section that best matches each baseline measuring site should be used for subsequent measurements.

(14) mRECIST1.1 recommends measuring all sites that have shrunk to less than the minimum measurable size (if measurable); if the tumor still exists but is too thin to measure, the default value is 2mm.

**Non-pleural lesions**

(15) "Measurable" non-pleural lesions require measurement of the longest diameter that represents up to 5 lesions (up to 2 lesions per organ) involved in all organs (minimum 10mm). The measurement of the pleural site is considered as a contribution of one organ, therefore, the measurement of the pleura does not exceed 2 (even if 6 are selected).

(16) Add all measurement values (pleural and non-pleural) to obtain a total measurement value.

(17) In the absence of measurable pleural disease, eligibility for clinical trials requires at least one measurable non-pleural lesion.

**Lymph nodes, unmeasurable pleural disease, measurable but unmeasured pleural disease, new lesions, or pleural thickening lesions**

(18) mRECIST1.1 includes lymph nodes in the total measurement and, where appropriate, accounts for the measurement values of RECIST1.1.

(19) Measurable but not used as a measurement site or pleural disease considered "unmeasurable" should be described. Unmeasurable pleural disease and measurable but unmeasured pleural disease can be combined into one description.

(20) According to mRECIST1.1, clear non-pleural new lesions or clear pleural thickening new lesions that exceed the minimum measurable size will be considered disease progression.

**Immunotherapy**

(21) mRECIST1.1 recommends using the iRECIST principle in immunotherapy trials where pseudo-progression and delayed response may be considered.

**Exclusions**

(22) Prior to prospective validation through clinical trials, mRECIST1.1 cannot recommend a different set of tumor response criteria for mesothelioma.

(23) mRECIST1.1 does not recommend incorporating tumor volume into current response criteria, but only as a research tool.

(24) mRECIST1.1 does not recommend using FDG-PET to evaluate treatment response in clinical trials.

**2 Measurement Guidelines for Pleural Mesothelioma (mRECIST1.1)**

**Measurement of Baseline Pleural Tumors and Other Sites**

1. Definition of Measurability

- If there is at least one pleural site with a tumor thickness ≥7mm, the pleural tumor is considered measurable.
- Non-pleural disease sites can be determined to be measurable, and the baseline one-dimensional maximum diameter is ≥10mm.
- The short diameter of the affected lymph nodes at baseline is ≥15mm.
- Pleural effusion is not considered a measurable lesion.

1. Baseline Measurement Sites

· The measurement of one-dimensional pleural tumors should construct a line segment perpendicular to the tangent of the pleural curve at the outer measurement point. The outer measurement point is preferably located on the chest wall or mediastinum to avoid anterior pleural reflection.

- The imaging section selected for measurement should be easily identifiable at subsequent time points and ideally related to clear and fixed mediastinal or chest wall anatomical landmarks.
- Avoid areas with lung collapse, pleural effusion, and blurred tumor borders as much as possible.
- If a measurement site is related to the pulmonary fissure, a one-dimensional measurement should be made perpendicular to the tangent of the pleura at that point.
- Record the thickness of the pleura at a maximum of two measurement sites, and if possible, record each independent axial section of the baseline chest CT scan (up to three). The axial sections should be at least 1cm apart. These (up to) six measurements added together are the total baseline pleural measurement.
- Each selected measurement must individually meet the minimum measurable disease criteria.
- If it is bilateral disease, the pleura is considered as one organ, and the pleural measurement sites can be distributed in two hemithoraces, with a maximum of three independent CT sections in each hemithorax, and no more than two measurement sites per section, for a total of six sites.
- One-dimensional non-pleural target lesion measurements that meet the measurable disease definition may include affected lymph node sites, chest wall masses or metastatic diseases, and their thickness measurements are added to the pleural tumor thickness measurement to generate the total baseline target lesion measurement. The measurement of non-pleural diseases can include up to five lesions, with no more than two lesions per organ. The measurement of pleural sites is considered a contribution of one organ.
- Other diseases appearing at baseline should be recorded as "non-target lesions." For diffuse pleural diseases, the description may include "widespread pleural thickening," "circumferential pleural thickening," "the tumor base cannot be distinguished from the diaphragm," "widespread pleural nodules," etc. This may include diseases that are not measurable and pleural diseases that are measurable but not measured. It is unrealistic to separately identify adjacent or excessive individual pleural lesions.
- Store images of the baseline scan measurement line segments for reference in subsequent image measurements.

**Disease measurement at follow-up time points**

(1) Pleural tumor measurements should always be taken at the same location and orientation as the baseline measurement, using the same image display parameters, regardless of changes in the shape or form of the lesion. If the tumor is still present but too thin to be measured on the magnified window, the default value of 2mm should be used.

(2) If a lesion observed on the same image plane at baseline appears best on a different image plane in subsequent scans due to chest contraction, breathing, or changes in patient position, it should be measured on the different image plane in subsequent scans.

(3) mRECIST1.1 recommends that the same observer measure all CT scans of a patient.

(4) Store the image of the baseline measurement line segment for reference when measuring subsequent time points.

**Definition of objective response**

(1) Complete response (CR): All pleural and non-pleural diseases disappear (including pleural thickening considered as a tumor).

(2) Partial response (PR): The total measurement value decreases by at least 30% compared to the baseline scan, and must be confirmed in at least four weeks on subsequent scans (the total measurement value should not exceed 70% of the baseline total measurement value).

(3) Disease progression (PD): The total measurement value increases by at least 20% compared to the previous minimum measurement value (including the baseline scan), even if the total measurement value is less than 70% of the baseline total measurement value. To evaluate PD, the absolute increase in the total measurement value must be at least 5mm. A clearly non-pleural new lesion or a clearly pleural thickening new lesion (representing a pleural tumor mass physically different from the existing measurement site) will be considered disease progression. "Clear" evaluation requires judgment and careful examination to ensure that the lesion has not appeared in the previous adjacent section.

(4) Stable disease (SD): The total measurement value decreases but does not reach PR, or increases but does not reach PD.

Note: Objective response must be confirmed at least four weeks later before being classified as confirmed PR or confirmed CR.

**Appendix - Medical treatment regimens**

The commonly used systemic treatment plan for malignant pleural mesothelioma (MPM) is shown in **Supplementary Table 2** as the first-line option. Other treatment plans are listed in **Supplementary Table 3**, and alternative options for certain situations are listed in **Supplementary Table 4**. For second-line systemic treatment plans for MPM, the preferred option is pemetrexed (recommended for patients who did not receive pemetrexed in first-line treatment; for patients who received first-line treatment with pemetrexed-containing regimens and experienced treatment failure, pemetrexed can still be used again, especially for young patients with good PS scores and long survival time without disease progression), followed by Nivolumab ± Ipilimumab (if not used in first-line treatment), Pembrolizumab, and other optional options such as gemcitabine and vinorelbine.

**Supplementary Table 2: shows the commonly used first-line systemic treatment plan for pleural mesothelioma.**

| Treatment regimen | Dosage and Administration | Treatment Cycle |
| --- | --- | --- |
| Pemetrexed  + Cisplatin | Pemetrexed 500mg/m^2^, intravenous drip, day 1  Cisplatin 75mg/m^2^, intravenous drip, day 1 and day 2, total dose is 75mg/m^2^ | Once every 3 weeks |
| Pemetrexed  + Cisplatin  + Bevacizumab | Pemetrexed 500mg/m^2^, intravenous drip, day 1  Cisplatin 75mg/m^2^, intravenous drip, day 1 and day 2, total dose is 75mg/m^2^  Bevacizumab 15mg/kg, intravenous drip, day 1 | Once every 3 weeks  6 cycles followed by bevacizumab maintenance therapy once every 3 weeks until disease progression |
| Nivolumab  + Ipilimumab | Nivolumab 360mg, intravenous drip, once every 3 weeks  Ipilimumab 1mg/kg, intravenous drip, once every 6 weeks | Until disease progression, intolerable adverse reactions, or 2 years of treatment. |

**Supplementary Table 3: Other commonly used treatment options for pleural mesothelioma.**

| Treatment regimen | Dosage and Administration | Treatment Cycle |
| --- | --- | --- |
| Pemetrexed  + Carboplatin  + Bevacizumab | Pemetrexed 500mg/m^2^, intravenous drip, day 1  Carboplatin, area under the curve (AUC) 5, intravenous drip, day 1  Bevacizumab 15mg/kg, intravenous drip, day 1 | Once every 3 weeks  6 cycles followed by bevacizumab maintenance therapy once every 3 weeks until disease progression |

**Supplementary Table 4: Useful in certain circumstances for pleural mesothelioma**

| Treatment regimen | Dosage and Administration | Treatment Cycle |
| --- | --- | --- |
| Gemcitabine  + Cisplatin | Gemcitabine 1000-1250mg/m^2^, intravenous drip, day 1, 8, and 15  Cisplatin 75mg/m2, intravenous drip, day 1 and day 2, total dose is 75mg/m2 | Once every 3 or 4 weeks |
| Pemetrexed  Vinorelbine | Pemetrexed 500mg/m^2^, intravenous drip, day 1  Vinorelbine 25-30mg/m^2^, intravenous drip, day 1 | Once every 3 weeks  Once every 1 week |

**Appendix - Comprehensive Treatment Principles**

(1) Comprehensive treatment principles for malignant pleural mesothelioma (MPM) that can be surgically resected: For patients with non-sarcomatoid mesothelioma (stages I-IIIA) that can be surgically resected, experienced thoracic surgeons should choose P/D or EPP to remove visible lesions, and intraoperative adjuvant chemotherapy and radiotherapy can be used. Postoperative adjuvant chemotherapy and half-chest IMRT are recommended for P/D, and postoperative adjuvant chemotherapy and half-chest radiotherapy are recommended for EPP. Postoperative prophylactic radiotherapy is not routinely recommended, but it can reduce the risk of surgical path conversion for patients who have not undergone adjuvant chemotherapy. The recommended radiotherapy dose is 45-60Gy/1.8-2Gy, and for patients with R2 resection, a dose of >60Gy can be used if adjacent tissue tolerance permits. The timing of postoperative adjuvant chemotherapy and radiotherapy should be determined by the MDT team.

(2) Comprehensive treatment principles for malignant pleural mesothelioma (MPM) that cannot be surgically resected: For patients with non-sarcomatoid mesothelioma (stages I-IIIA) that cannot be surgically resected, neoadjuvant chemotherapy can be attempted followed by surgical treatment and sequential postoperative radiotherapy. For patients who cannot undergo surgery, systemic chemotherapy can be performed, and the treatment principles and regimens are described in the internal medicine treatment section. For patients with stage IIIB, stage IV, or sarcomatoid mesothelioma, or malignant pleural mesothelioma (MPM) that is not suitable for surgery, systemic chemotherapy or supportive treatment can be performed depending on the patient's general condition.

[1] Tsao AS, Garland L, Redman R et al. A practical guide of the Southwest Oncology Group to measure malignant pleural mesothelioma tumors by RECIST and modified RECIST criteria. J Thorac Oncol. 2011; 6(3): 598-601.

[2] Armato SG 3rd, Nowak AK. Revised Modified Response Evaluation Criteria in Solid Tumors for Assessment of Response in Malignant Pleural Mesothelioma (Version 1.1). J Thorac Oncol. 2018; 13(7):1012-1021.
